# Vertical Graphene-Supported NiMo Nanoparticles as Efficient Electrocatalysts for Hydrogen Evolution Reaction under Alkaline Conditions

**DOI:** 10.3390/ma16083171

**Published:** 2023-04-18

**Authors:** Hongbin Wang, Beirong Ye, Chen Li, Tao Tang, Sipu Li, Shaojun Shi, Chunyang Wu, Yongqi Zhang

**Affiliations:** 1 Institute of Fundamental and Frontier Sciences, University of Electronic Science and Technology of China, Chengdu 611731, China; 2 Jiangsu Laboratory of Advanced Functional Material, School of Chemistry and Materials Engineering, Changshu Institute of Technology, Changshu 215500, China; 3 State Key Laboratory of Electronic Thin Film and Integrated Devices, University of Electronic Science and Technology of China, Chengdu 610054, China

**Keywords:** vertical graphene, NiMo alloy, high surface area, hydrogen evolution reaction, electrodeposition

## Abstract

Water electrolysis as an important and facile strategy to generate hydrogen has attracted great attention, and efficient electrocatalysts play a key role in hydrogen evolution reaction (HER). Herein, vertical graphene (VG)-supported ultrafine NiMo alloy nanoparticles (NiMo@VG@CC) were fabricated successfully via electro-depositing as efficient self-supported electrocatalysts for HER. The introduction of metal Mo optimized the catalytic activity of transition metal Ni. In addition, VG arrays as the three-dimensional (3D) conductive scaffold not only ensured high electron conductivity and robust structural stability, but also endowed the self-supported electrode large specific surface area and exposed more active sites. With the synergistic effect between NiMo alloys and VG, the optimized NiMo@VG@CC electrode exhibited a low overpotential of 70.95 mV at 10 mA cm^−2^ and a remarkable stable performance over 24 h. This research is anticipated to offer a powerful strategy for the fabrication of high-performance hydrogen evolution catalysts.

## 1. Introduction

The highest energy density and zero carbons emissions cause the hydrogen (H_2_) economy to develop rapidly [1,2,3,4]. Currently, hydrogen production comes almost entirely from fossil fuels. In order to produce adequate H_2_, 2% of coal and 6% of natural gas are consumed, which results in annual emissions of around 830 million tons of carbon dioxide, which has a significant greenhouse effect. From this perspective, splitting water via electrocatalysis based on renewable energy to get clean H_2_ and oxygen is an attractive alternative strategy [5,6]. The sluggish reaction process of water splitting makes a high overpotential required, which leads to a low energy conversion efficiency. Therefore, highly efficient catalysts are indispensable to accelerate the water splitting process. Until now, noble metal-based materials, such as Pt, have delivered the best catalytic activity and have acted as the benchmark of HER electrocatalysts. However, scarce property and high cost hinders their extensive application. Consequently, there have been significant efforts made to investigate the highly active, inexpensive, and abundant non-noble metal alternatives, such as transition metals and their alloys/compounds [7,8,9,10,11,12,13,14] and carbon-based materials [15].

Ni metal and its alloys foils have been extensively applied in the industrial H_2_ production as the anode of water splitting. Among them, NiMo-based catalysts have been explored as effective HER catalysts with high electrochemical activity due to the synergistic interactions between Ni and Mo [16,17,18]. To increase the metal utilization and improve the catalytic activity of NiMo alloy catalysts, constructing nanosized catalysts is a feasible strategy and has been studied extensively. For instance, Feng and coworkers fabricated MoO_2_ cuboid-supported MoNi_4_ nanoparticles via an outward diffusion strategy on annealing NiMoO_4_ cuboids on Ni foam (MoNi_4_/MoO_2_@Ni) [19]. The high specific surface area and ordered nanostructure of MoNi_4_/MoO_2_@Ni endows its almost zero onset overpotential. Ito and co-workers created non-noble-metal electrodes by a bi-continuous and open-pored NiMo alloy which was embodied in nitrogen-doped (N-doped) holey graphene with nanoscale openings [20]. Hu and co-workers synthesized HER catalysts with excellent stability and high performance in acidic condition by synthesizing three-dimensional N-doped graphene (3DNG)-supported NiMo NPs enclosed by N-doped graphene sheet layers (NGLs), obtaining a performance comparable to that of Pt because of the high electrical conductivity/electron flow [21]. These literatures confirm that constructing multilevel nanostructured electrodes with innately catalytically active surface species by simple strategy is an effective means to obtain catalysts with high HER performance [22,23]. 

Vertical graphene (VG) array film, one of the 3D-oriented arrangement carbon materials, has drawn attention as the ideal 3D scaffold due to the following inherent physical and chemical characteristics, such as abundant edges, open channels, large surface, mechanical stability, excellent conductivity, and robust connection. Recently, VG has been applied in different fields [24,25,26,27,28,29,30,31]. For example, VG-supported MoS_2_ nanosheets were synthesized via hydrothermal reaction and exhibited outstanding HER electrocatalytic performance, with an overpotential at 10 mA cm^−2^ (η_10_) of 78 mV [32]. Tsounis and co-workers synthesized the edge-rich vertical graphene (er-VG)-supported Ni–Fe hydroxide and applied for oxygen evolution reaction (OER). The composite electrode with er-VG as support delivered better catalytic activity (an overpotential of 276 mV to reach 10 mA cm^−2^) than that of Ni-Fe hydroxide catalysts on different supports (Cu foam, Ni foam, and graphene paper) [33].

Herein, we successfully fabricated NiMo@VG catalysts on carbon cloth substrate (NiMo@VG@CC) through a simple two-step process and directly acted as a self-supported electrocatalysis for HER. The in-situ growth of active materials on the substrate slashes the impedance to charge transfer while boosting mechanical stability, which can boost the practical catalytic performances of the electrode to some extent [34,35]. Moreover, VG with high surface area and high conductivity can augment the number of active sites for deposition NiMo alloys and further contribute to the fast transmission of electrons during the HER process. Through rationally designing the morphology, hydrophily, and the structural characteristics, the optimized NiMo@VG@CC exhibited a low overpotential of 70.95 mV at 10 mA·cm^−2^. The results show unequivocally that well-designed 3D VG coupled NiMo-based electrocatalysts have a great deal of promise for extremely effective electrocatalytic HER, presenting a new path towards substituting a wider range of alternatives for noble metals in a wide range of applications.

## 2. Materials and Methods

### 2.1. Materials and Regents

Sodium molybdate dihydrate (Na_2_MoO_4_·2H_2_O), sodium citrate tribasic hydrate (Na_3_C_6_H_5_O_7_·nH_2_O), and ammonia (25% NH_3_) were purchased from Shanghai Aladdin Biochemical Technology Co., Ltd., Shanghai, China. Nickel sulfate hexahydrate (NiSO_4_·6H_2_O), potassium hydroxide (KOH), and 20 wt.% Pt/C were purchased from Shanghai Macklin Biochemical Co., Ltd., Shanghai, China. CH_4_ (purity: 99.999%), H_2_ (purity: 99.999%), and Ar (purity: 99.999%) were purchased from Date Gas Co., Ltd., Chengdu, China. Carbon cloth (CC) was bought from Taiwan Ce Tech Co., Ltd., Taiwan, China. Sulfuric acid (H_2_SO_4_, 95.0~98.0%) and nitric acid (HNO_3_, 65.0~68.0%) were purchased from Sinopharm Chemical Reagent Co., Ltd., Shanghai, China. The deionized water was produced by the Milli-Q system (18 MΩ cm^−1^). All agents and solvents were of analytical grade and were used without any further purification.

### 2.2. Synthetic of VG@CC

VG was grown in a home-made PECVD device. Before synthesizing VG, the PECVD device was preheated to about 500 °C. Following that, CC (20 mm × 50 mm) as the substrate was positioned in the center of the PECVD system. The atmosphere inside of the device was evacuated until the pressure was lower than 50 Pa. After introducing CH_4_ (6 sccm), H_2_ (10 sccm), and Ar (20 sccm) into the system, 500 W of radio frequency source power was used for generating plasma, lasting 8 min. Here, for VG growth, the CH_4_ was used as the carbon source. The VG@CC was obtained once the system reached room temperature. Two different methods were used for the hydrophilic treatment of VG@CC. One was the acid treatment of VG@CC, that is, heating in mixed acid (H_2_SO_4_ and HNO_3_ with a volume ratio of 1:3) at 60 °C for 4 h. The other was treating the VG@CC in a vacuum (~20 Pa) for 10 s at 100 W power using plasma technology. VG@CC treated by acid and plasma technology was named as VG@CC-AC and VG@CC-PL, respectively. Moreover, CC was also hydrophilic treatment for further use, and was named as CC-AC and CC-PL, respectively.

### 2.3. Synthetic of NiMo@VG@CC

The ultrafine NiMo was deposited via a pulse electrodeposition technique (Shanghai Chenhua Instrument Co., Ltd., Shanghai, China). Pt foil served as the counter-electrode and VG@CC (VG@CC-AC and VG@CC-PL) served as the work electrode, respectively. 6 mM Na_3_C_6_H_5_O_7_ nH_2_O, 40 mM NiSO_4_ 6H_2_O, 60 mM Na_2_MoO_4_, and 200 mL H_2_O made up the deposition solution. The pH was then raised to 10 using ammonia. Deposition was carried out at 25 °C with a cathodic current density of 10 mA cm^−2^ and a frequency of 2 Hz with different cycles (500, 1700, 2900, 4000, 4100, and 5300). Samples were washed and submerged in DI water for at least 5 min to eliminate electrolyte residue. After that, the final products were dried at 60 °C for at least 8 h. NiMo on VG@CC-AC and VG@CC-PL were named as NiMo@VG@CC-AC and NiMo@VG@CC-PL, respectively. In addition, NiMo on CC-AC and CC-PL were also fabricated as the counterpart samples. The mass loading of NiMo in VG@CC-AC, VG@CC-PL, CC-AC, and CC-PL was around 1 mg/cm^2^, respectively. 

### 2.4. Synthetic of Ni@VG@CC-AC

The ultrafine Ni was deposited via a pulse electrodeposition technique (Shanghai Chenhua Instrument Co., Ltd., Shanghai, China). Pt foil served as the counter-electrode and VG@CC-AC served as the work electrode, respectively. 6 mM Na_3_C_6_H_5_O_7_ nH_2_O, 40 mM NiSO_4_ 6H_2_O, and 200 mL H_2_O made up the deposition solution. The pH was then raised to 10 using ammonia. Deposition was carried out at 25 °C with a cathodic current density of 10 mA cm^−2^ and a frequency of 2 Hz with 4000 cycles. Samples were washed and submerged in DI water for at least 5 min to eliminate electrolyte residue. After that, the final products were dried at 60 °C for at least 8 h and named as Ni@VG@CC-AC. The mass loading of Ni in VG@CC-AC was around 1 mg/cm^2^, respectively. 

### 2.5. Synthetic of Mo@VG@CC-AC

The ultrafine Mo was deposited via a pulse electrodeposition technique (Shanghai Chenhua Instrument Co., Ltd., Shanghai, China). Pt foil served as the counter-electrode and VG@CC-AC served as the work electrode, respectively. 6 mM Na_3_C_6_H_5_O_7_ nH_2_O, 60 mM Na_2_MoO_4_, and 200 mL H_2_O made up the deposition solution. The pH was then raised to 10 using ammonia. The deposition was carried out at 25 °C with a cathodic current density of 10 mA cm^−2^ and a frequency of 2 Hz with 4000 cycles. Samples were washed and submerged in DI water for at least 5 min to eliminate electrolyte residue. After that, the final products were dried at 60 °C for at least 8 h and named as Mo@VG@CC-AC. The mass loading of Mo in VG@CC-AC was around 1 mg/cm^2^, respectively. 

### 2.6. Characterization

To ascertain the crystal structures of the samples, an X-ray diffraction measurement (XRD, Shimadzu ZD-3AX diffractometer, Shimadzu Co., Ltd., Kyoto, Japan) was performed. The micro-nano morphology of the materials was examined using a scanning electron microscope (SEM, Hitachi S-4700, Hitachi High-Tech Co., Ltd., Tokyo, Japan) and a transmission electron microscope (TEM, FEI Tecnai G2 F20, Thermo Fisher Scientific Inc., Waltham, MA, USA). The surface elements and related valence states of the samples were further confirmed using X-ray photoelectron spectroscopy (XPS, K-alpha, Thermo Fisher Scientific Inc., Waltham, MA, USA). The element composition of the NiMo@VG@CC-AC was quantitatively assessed using energy-dispersive X-ray spectroscopy (EDS) analysis with a high probe current of 10 Å.

### 2.7. Electrochemical Measurements

All electrochemical tests were carried out on a CHI 760E electrochemical workstation in 1.0 M KOH at 25 °C, including cyclic voltammetry (CV), linear sweep voltammetry (LSV), electrochemical impedance spectroscopy (EIS), and chronopotentiometry (CP). Obtained samples, graphite rod (diameter: 5 mm, length: 2 cm), and Ag/AgCl (3.0 M KCl) electrode were served as the working, counter, and reference electrodes, respectively, in a conventional three-electrode configuration. The LSV tests were carried out at a scan rate of 5 mV s^−1^. EIS measurements were tested between 10^5^ and 0.5 Hz. The electrochemical double-layer capacitance (C_dl_) was utilized to measure the electrochemical specific surface area (ESEA) via a CV test at various scan speeds between 4 and 20 mV s^−1^. A stability test was carried out at a constant current density of 10 mA cm^−2^ for 24 h. The Nernst equation was used to adjust the potential scale with regard to relative hydrogen electrode (RHE), where $E_{vs.\;RHE}=E_{vs.\;Ag/AgCl}+0.0592\times pH+0.197\;V$. Unless otherwise specified, all measurements were iR-corrected.

## 3. Results and Discussion

**Figure 1:**
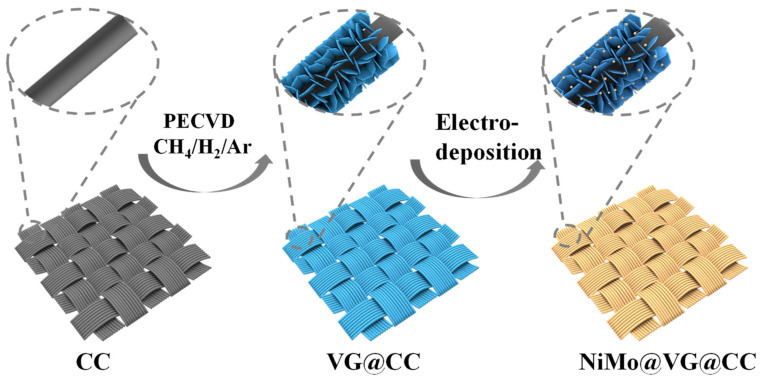
Schematic of the synthesis process of NiMo@VG@CC.

**Figure 2:**
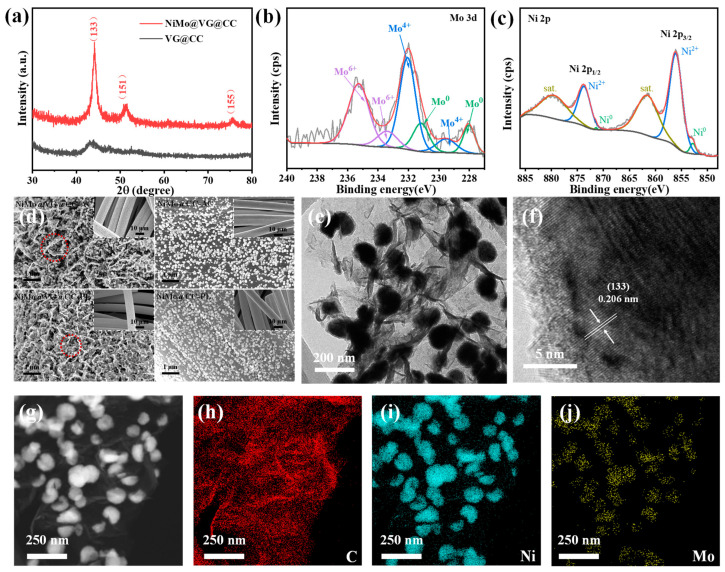
Structure and morphology characterizations of NiMo@VG@CC-AC. (**a**) XRD patterns; (**b**,**c**) High-resolution XPS spectra of Mo 3d and Ni 2p; (**d**) SEM; (**e**,**f**) TEM; (**g**–**j**) EDS mapping of C, Ni, and Mo.

The overall synthesis process of NiMo@VG@CC is illustrated in Figure 1. In brief, by using CC as a substrate, VG was firstly prepared by the PECVD method. There are three main stages in the growth of VG. (i) Nucleation, random fissures, and suspended bonds serve as nucleation sites on the surface of carbon fabric and create a buffer layer. (ii) For growth, carbon atoms were continually attached to the opening edge of the VG sheets when graphene nano-sheets grew vertically under the effect of stress and/or a local electric field. (iii) Termination—when the opening edge is closed, the growth of VG eventually stops due to competition between material deposition and etching action in the plasma [36]. Following that, NiMo alloy nanoparticles were uniformly decorated on the surface of the VG via a pulse electrodeposition process. Due to the different atomic radius of the nickel (0.124 nm) and molybdenum (0.140 nm), their co-deposition behavior can also be referred to as induced co-deposition of molybdenum [37]. The crystal structure of obtained VG@CC-AC and NiMo@VG@CC-AC were examined using the XRD. As shown in Figure 2a, VG@CC-AC (black line) and NiMo@VG@CC-AC (red line) showed a different form. Peaks at 43.9°, 52.1°, and 75.5° in NiMo@VG@CC-AC were indexed to the (133), (151), and (155) lattice planes of NiMo alloy (PDF#48-1745), confirming the successful formation of NiMo alloys on VG@CC-AC. 

The full XPS spectra (Figure S1) amply demonstrates that the NiMo@VG@CC-AC is entirely made up of the components C, O, Ni, and Mo with no extra impurities. The surface oxidation of the sample caused by air exposure may be the source of the O elements. The deconvolution of Mo 3d spectra of NiMo@VG@CC-AC resulted in three spin-orbit doublets (Figure 2b). The binding energies at ~227.6 and 230.7 eV belonged to Mo^0^ 3d_5/2_ and Mo^0^ d_3/2_, respectively, indicating the presence of metallic Mo [38,39]. XPS binding energy region is in accordance with Ni 2p spectrum of NiMo@VG@CC-AC (Figure 2c) and can be decomposed into two signals at ~869.7 and 870.6 eV in accordance with NiMo (2p^1/2^) and NiO (2p^1/2^), respectively [16,40]. The inevitable surface oxidation in the atmospheric environment is what leads to the oxidation state of metal atoms [41].

The micro-nano morphologies of the sample were systematically analyzed through SEM. The disordered arrangement of pure VG grown on CC forms a honeycomb structure, and the domain size of ~100 nanometers (Figure S2). Figure 2d and the insets depict the morphological characteristics of NiMo alloys (~50–150 nm) deposited on different substrates, including CC-AC, CC-PL, VG@CC-AC, VG@CC-PL. Obviously, VG can serve as a supporter to deposit NiMo alloys, which leads to a totally different morphologies between CC substrate with and without VG. Many active sites for the deposition of NiMo alloys are provided by the homogenous honeycomb-like graphene nanowalls that were created on the substrates by randomly oriented graphene sheets. The low-magnification SEM images (insets in Figure 2d) exhibit the homogenous distribution of the products. Moreover, the morphology of NiMo@VG@CC-AC shows many more holes compared with NiMo@VG@CC-PL, indicating its preferable wettability of electrolyte, which is conducive to the HER process. Therefore, VG@CC-AC was selected for further study. The SEM images of NiMo deposited on VG@CC-AC with different deposition cycles are shown in Figure S3. As can be seen, with the increase of the deposition cycles from 500 to 4100 (Figure S3a–d), the size of the NiMo alloys grown on the surface of VG@CC-AC is gradually increasing. When the deposition cycles continue to increase to 5300, the pore structure of the VG@CC-AC is blocked by the growing NiMo alloy (Figure S3e), which leads to the active materials in the electrode not being fully utilized. The performance of the electrode’s HER can be considerably impacted by the number of holes and electrochemically active spots. Overall, 4100 cycle depositions were chosen as the optimum deposition condition for synthesize of NiMo alloys on VG@CC-AC. The SEM results indicate that the homogenous nanostructure of NiMo-VG@CC samples is mostly affected by the substrate’s structure and the deposition cycle.

The TEM image (Figure 2e) revealed that the NiMo alloyed metal nanoparticles on the VG nanosheets were distributed uniformly with a 100 nm diameter. The magnified TEM picture in Figure 2f reveals the interplanar spacings of 0.206 nm for the corresponding (133) planes of NiMo alloys. Meanwhile, Figure 2g–j and Figure S4 depict the elemental distribution of the NiMo@VG@CC-AC hybrids. The cohabitation of C, Ni, and Mo in the hybrids is evident from the apparent homogeneous distribution of the C, Ni, and Mo components in the NiMo@VG@CC-AC. However, the concentration of Mo (14%) in NiMo@VG@CC-AC is lower than that of Ni (86%), which is mainly because Ni is easier to be deposited in aqueous solution than Mo is [42].

**Figure 3:**
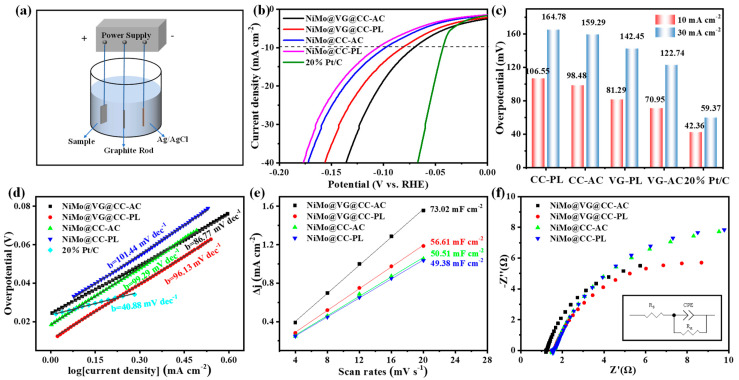
Electrochemical performance of composite catalysts in 1 M KOH electrolyte. (**a**) Three-electrode set; (**b**) HER polarization curves; (**c**) Comparison of overpotential at the 10 mA cm^− 2^ and 30 mA cm^− 2^; (**d**) Corresponding Tafel plots; (**e**) Calculated double layer capacitance (C_dl_) values; (**f**) Nyquist plots at the overpotential of 80 mV.

The catalytic HER activities of the catalysts were tested in 1.0 M KOH solution through a three-electrode system, as illustrated in Figure 3a. Prior to comparing the catalyst performance of NiMo@CC-AC, NiMo@CC-PL, NiMo@VG@CC-AC, NiMo@VG@CC-PL and 20 wt.% Pt/C electrode, the optimized NiMo@VG@CC-AC electrocatalysts were created by altering their deposition cycles to achieve the optimum electrochemical performance (Figures S5–S7 and Table S1). With the increase of deposition cycles from 500 to 4100, the HER properties of NiMo@VG@CC-AC are gradually improved. When the deposition cycles increased to 5300, the electrocatalytic performance of the NiMo@VG@CC-AC electrode is basically similar to that of 4100. The optimized deposition can be set as 4100 cycles, which is in accordance with the SEM test results. In addition, under the same substrate conditions, the electrochemical performance of monometallic and bimetallic catalysts was also compared for verifying the excellent performance conjecture of the alloy. According to Figures S8–S10 and Table S2, it can indeed show that the catalytic activity of NiMo@VG@CC-AC is significantly better than that of Ni@VG@CC-AC and far better than that of Mo@VG@CC-AC, and it is obvious that the alloy strategy plays a crucial role in the improvement of HER performance. Figure 3b displays the optimized NiMo@VG@CC-AC, NiMo@CC-AC, NiMo@CC-PL, NiMo@VG@CC-AC, and 20% Pt/C electrode. Obviously, VG on CC has a positive ongoing impact on NiMo’s HER performance. This is mainly because the existence of VG constructed a three-dimensional (3D) conductive scaffold between NiMo alloys and CC, which not only guarantees strong electron conductivity and structural stability, but also provides the rough surface necessary for securely grabbing the following NiMo alloys. At the same time, hydrophilic treatment of VG@CC by different methods also has an important bearing on the performance of HER, which is mainly due to, in contrast with acid treatment, the high power of plasma treatment is ease to destroy the original micro-structures of VG (Figure 2d), such as the decreased pore structure, which is unfavorable for the deposition of NiMo alloys and further decreases the active sites for HER. Therefore, at a current density of 10 mA cm^−2^, the overpotential of each catalyst ranked in the following order: 20% Pt/C (42.36 mV) < NiMo@VG@CC-AC (70.95 mV) < NiMo@VG@CC-PL (81.29 mV) < NiMo@CC-AC (98.48) < NiMo@CC-PL (106.5 mV) (Figure 3c). In addition, we also compared the HER catalytic performance of various NiMo-based catalysts on different substrates, and it can be seen that the HER performance of the prepared NiMo@VG@CC-AC is superior to most catalysts (Supporting Information Table S3).

Figure 3d displays the matching Tafel plots to assess the HER process catalysts’ kinetics of electron transfer. NiMo@VG@CC-AC has a smaller Tafel slope than NiMo@VG@CC-PL (86.77 mV dec^−1^), NiMo@CC-AC (99.29 mV dec^−1^) and NiMo@CC-PL (101.44 mV dec^−1^), showing that the NiMo@VG@CC-AC has a faster electron transfer process. The introduction of VG improved the performance of the NiMo catalysts to a certain extent, manifesting that NiMo alloys’ electrochemical performances can be enhanced by VG via improving their kinetic HER process. 

The high specific surface area NiMo@VG@CC-AC electrode’s 3D hierarchical structure creates a large number of easily available metal sites, which is advantageous for accelerating the HER process. In 1 M KOH electrolyte, we performed cyclic voltammetry tests on catalysts with different substrates at different scan rates of 4, 8, 12, 16, and 20 mV s^−1^, the results obtained are shown in Figure S11, and the calculation of C_dl_ in the non-faradaic area of the CV was used to establish the ECSA of all samples. Figure 3e demonstrates that the NiMo@VG@CC-AC has the highest ECSA value (73.02 mF cm^−2^), which is roughly 1.2 to 1.5 times greater than that of the NiMo@VG@CC-PL (56.61 mF cm^−2^), NiMo@CC-AC (49.38 mF cm^−2^), and NiMo@CC-PL (50.51 mF cm^−2^). To further understand the HER dynamics taking place in the electrode/electrolyte interface, EIS measurements were performed at an AC voltage of 80 mV (Figure 3f). Two distinct frequency regimes are clearly visible in the Nyquist plots—one in the low frequency range and the other in the high frequency region. It was revealed that the NiMo@VG@CC-AC electrode appears to have lower charge-transfer resistance than the NiMo-based electrode, which suggests that it has a higher capacity for charge-transfer during the HER process [43]. Associated EIS data with equivalent circuit diagrams and more detailed simulations were placed in Figure S12 and Table S4. The 3D hierarchical porous structure constructed by introducing of VG nanosheets provide NiMo alloys and CC substrate a highly conductive surface and strong electron interaction, which ensures minimum charge-transfer resistance of NiMo@VG@CC-AC electrode and causes electrode/electrolyte contact with a high electron transfer rate. 

**Figure 4:**
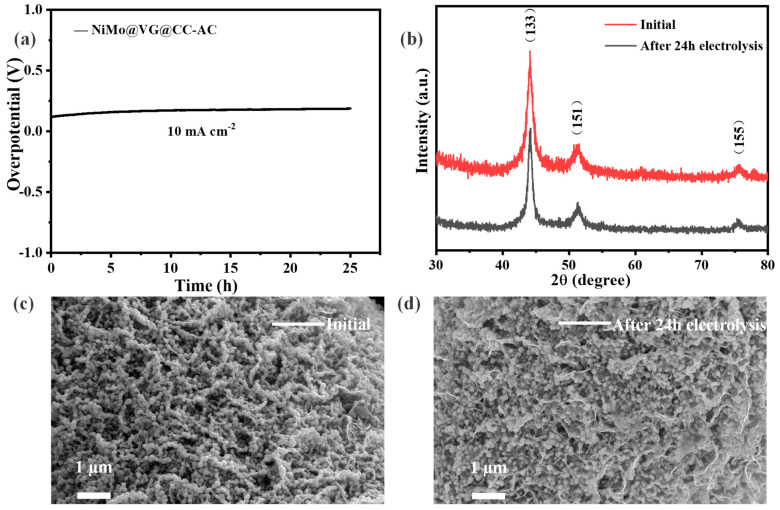
Stability test of NiMo@VG@CC-AC at 10 mA cm^−2^ for 24 h. (**a**) Compared LSV polarization curves at the scan rate of 5 mV s^−1^ and long-term cycling stability performance, (**b**) Compared XRD patterns initial and after 24 h electrolysis, SEM images of NiMo@VG@CC-AC initial (**c**) and after (**d**) stability test.

Long-term stability is another important aspect to evaluate HER catalysts in addition to their catalytic activity. The stability of the NiMo@VG@CC-AC was assessed for 24 h at 10 mA cm^−2^ of constant current density at room temperature. According to Figure 4a, the overpotential of NiMo@VG@CC-AC electrode remained almost steady during the whole testing period, demonstrating its good durability. Figure 4b shows that the XRD data of NiMo@VG@CC-AC before and after the stability test have almost no change, which means that the crystal structure of the synthetic material is well-maintained. Meanwhile, the SEM measurements were measured to further investigate the causes of stability degradation of NiMo@VG@CC-AC electrode. As shown in Figure 4c,d, although there is no significant structural distortion of the tested electrode, the partial peeling of NiMo alloys can be seen on some deposition spot, which may lead to the decreased durability of the catalyst. 

## 4. Conclusions

In conclusion, the NiMo@VG@CC hybrid catalytic electrode, which directly acted as a self-supported electrode for alkaline water splitting, was effectively synthesized via PECVD and the following electrodeposition procedure. Comparative studies showed that the distinctive characteristics of VG endowed the electrode with a 3D hierarchical porous structure and a high electrochemical specific surface area, which benefits the diffusion of the generated gas and prevents the agglomeration of the active materials, thereby improving the alkaline HER performance of the NiMo@VG@CC-AC electrode. The prepared NiMo@VG@CC-AC electrode had impressive electrocatalytic performance at 1 M KOH electrolyte with a low HER overpotential of 70.95 mV at 10 mA cm^−2^, which is superior to most NiMo-based catalysts on various substrates under the same experimental conditions and satisfying stability under low current density over 24 h. This work is expected to provide theoretical reference for the deposition of alloys on vertical graphene and the regulation of catalytic hydrogen evolution performance.

## Data Availability

The data presented in this study are available on request from the corresponding author.

## Supplementary Materials

The following supporting information can be downloaded at: https://www.mdpi.com/article/10.3390/ma16083171/s1, Figure S1: The wide-scan XPS spectra of NiMo@VG@CC-AC; Figure S2: SEM image of VG@CC; Figure S3: SEM images of NiMo@VG@CC-AC at the different deposition cycles; Figure S4: EDS pattern of NiMo@VG@CC-AC; Figure S5: HER performance of NiMo@VG@CC-AC at the different deposition cycles in 1 M KOH electrolyte; Figure S6: Cyclic voltammogram curves in double layer region at scan rates of 4, 8, 12, 16, 20 mV·s^−1^ of NiMo@VG@CC-AC at the different number of deposition cycles in 1 M KOH electrolyte; Figure S7: Nyquist plots at the overpotential of 10 mV of NiMo@VG@CC-AC at the different number of deposition cycles in 1 M KOH electrolyte; Figure S8: Electrochemical performance of composite catalysts in 1 M KOH electrolyte; Figure S9: Cyclic voltammogram curves in double layer region at scan rates of 4, 8, 12, 16, 20 mV·s^−1^ of composite catalysts in 1 M KOH electrolyte; Figure S10: Nyquist plots of composite catalysts in 1 M KOH electrolyte; Figure S11: Cyclic voltammogram curves in double layer region at scan rates of 4, 8, 12, 16, 20 mV·s^−1^ of composite catalysts in 1 M KOH electrolyte; Figure S12: Nyquist plots of composite catalysts in 1 M KOH electrolyte. Table S1: Impedance circuit diagram fitting parameters of NiMo@VG@CC-AC with different deposition cycles; Table S2: Impedance circuit diagram fitting parameters of different composite catalysts; Table S3: Performance comparison of NiMo-based catalysts on various substrates; Table S4: Impedance circuit diagram fitting parameters of NiMo alloy catalysts on various substrates. References [44,45,46,47,48,49,50,51,52,53,54,55] are cited in the supplementary materials.
