# Supplementary material for: Vertical Graphene-Supported NiMo Nanoparticles as Efficient Electrocatalysts for Hydrogen Evolution Reaction under Alkaline Conditions
Source: Materials (Basel). 2023 Apr 18;16(8):3171. doi: 10.3390/ma16083171 (PMC10145461; doi:10.3390/ma16083171)
Supplement: Supplementary file 1 [file materials-16-03171-s001.zip › materials-2281035-supplementary.pdf]

Supplementary Materials

Vertical Graphene-Supported NiMo Nanoparticles as Efficient Electrocatalysts for Hydrogen Evolution Reaction under Alkaline Conditions

Hongbin Wang [1], Beirong Ye [1,*], Chen Li [1], Tao Tang [1], Sipu Li [1], Shaojun Shi [2], Chunyang Wu [3] and Yongqi Zhang [1,*]

[1] Institute of Fundamental and Frontier Sciences, University of Electronic Science and Technology of China, Chengdu 611731, China
[2] Jiangsu Laboratory of Advanced Functional Material, School of Chemistry and Materials Engineering, Changshu Institute of Technology, Changshu 215500, China
[3] State Key Laboratory of Electronic Thin Film and Integrated Devices, University of Electronic Science and Technology of China, Chengdu 610054, China
* Correspondence: yebeirong@uestc.edu.cn (B.Y.); yqzhang@uestc.edu.cn (Y.Z.)

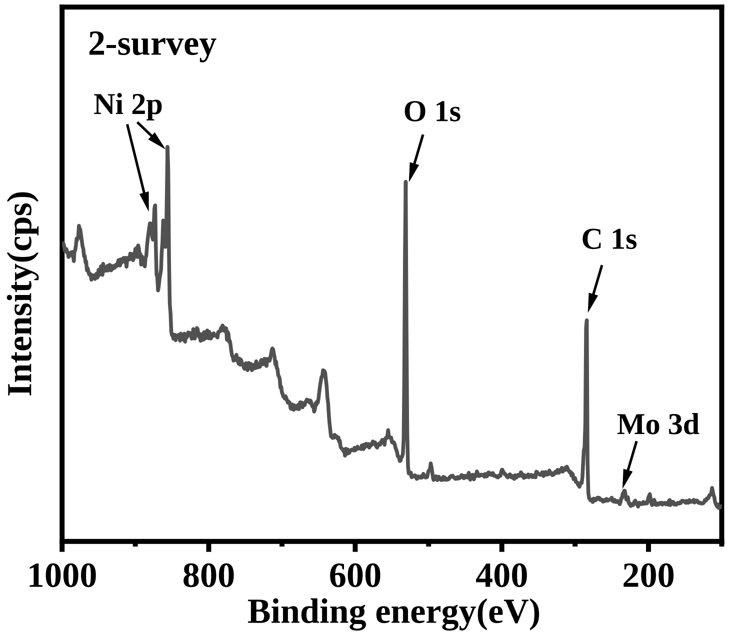

Figure S1. The wide-scan XPS spectra of NiMo@VG@CC-AC.

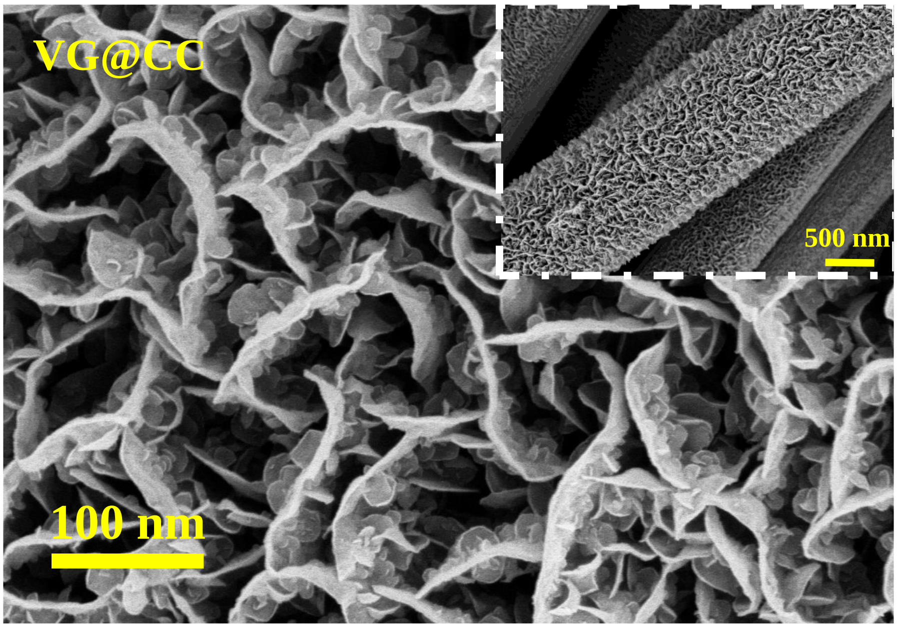

Figure S2. SEM image of VG@CC.

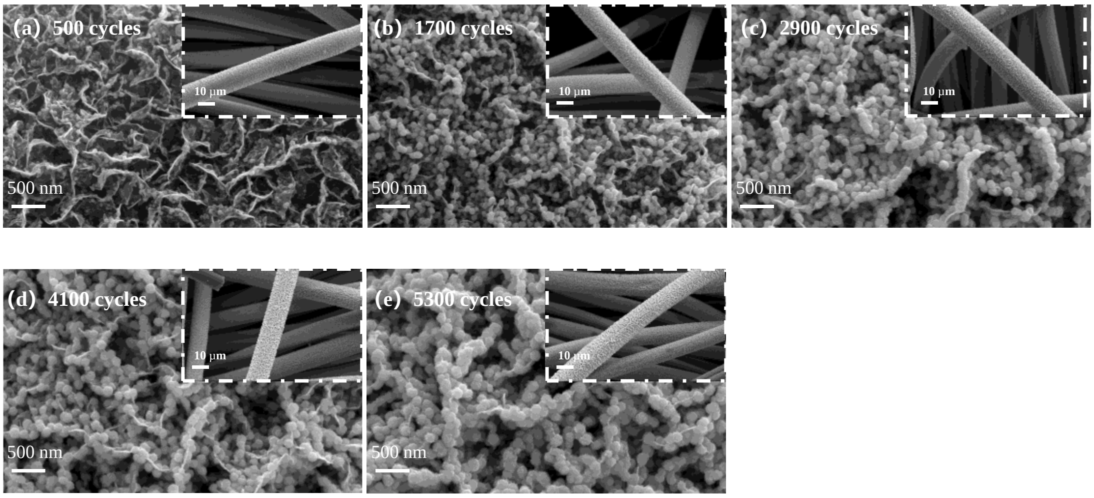

Figure S3. SEM images of NiMo@VG@CC-AC at the different deposition cycles. (a) 500 cycles. (b) 1700 cycles. (c) 2900 cycles. (d) 4100 cycles. (e) 5300 cycles.

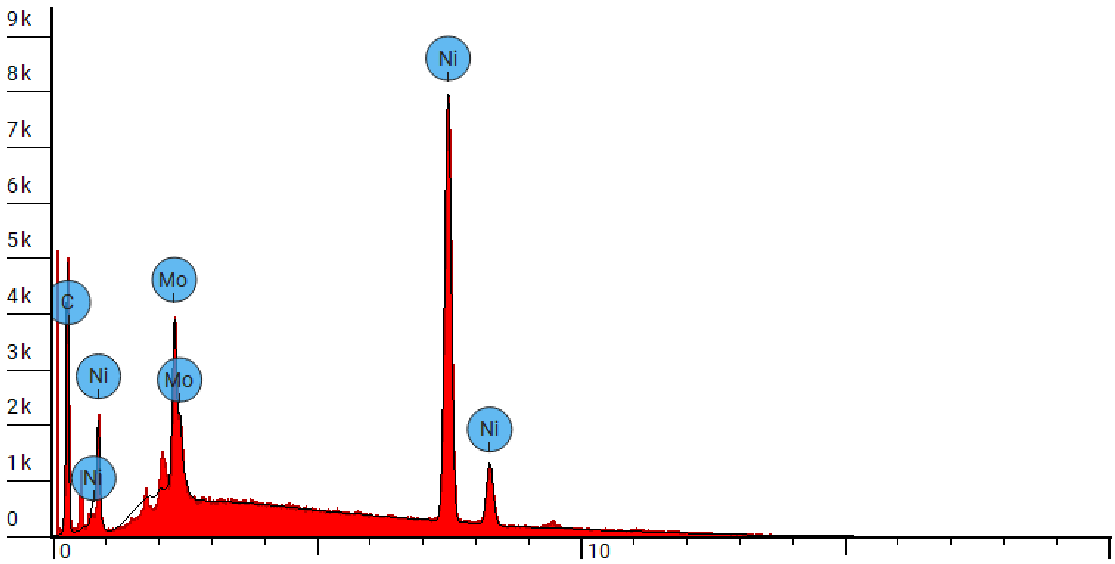

Element Number	Element Symbol	Element Name	Atomic Conc.	Weight Conc.
28	Ni	Nickel	86.004	79.984
42	Mo	Molybdenum	13.996	20.016

Figure S4. EDS pattern of NiMo@VG@CC-AC.

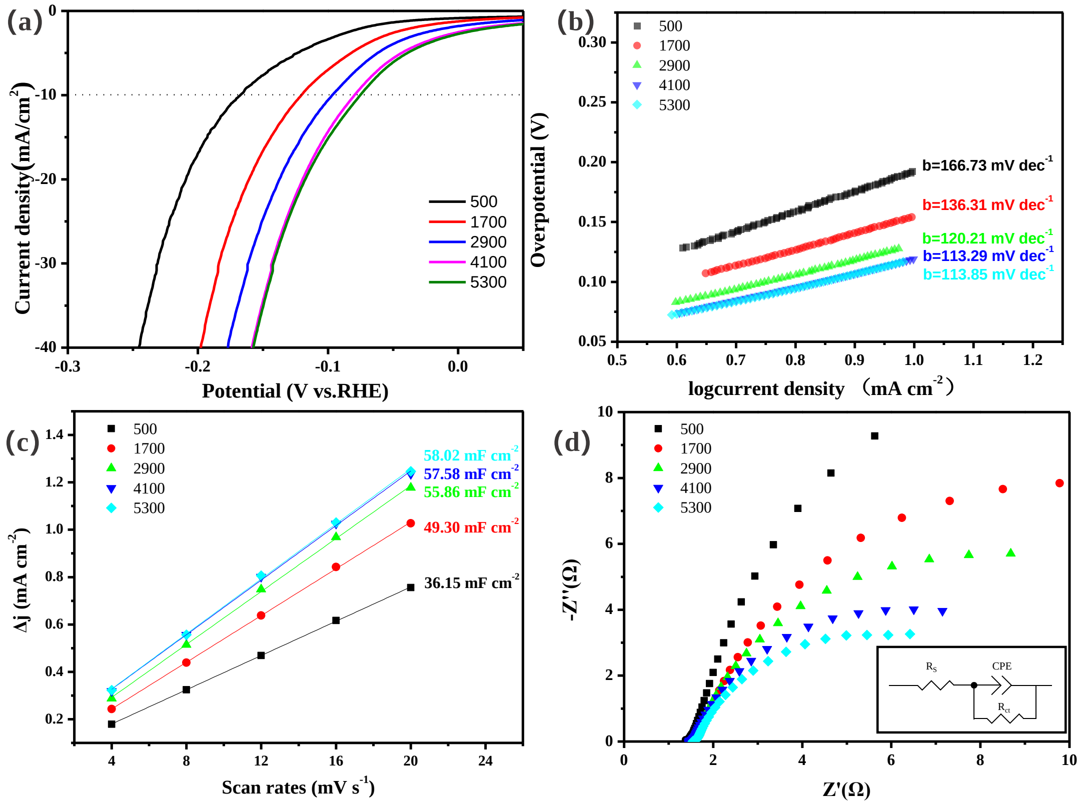

Figure S5. HER performance of NiMo@VG@CC-AC at the different deposition cycles in 1 M KOH

electrolyte. (a) HER polarization curves and (b) corresponding Tafel plots, (c) calculated double layer capacitance (C_{dl}) values, (d) Nyquist plots.

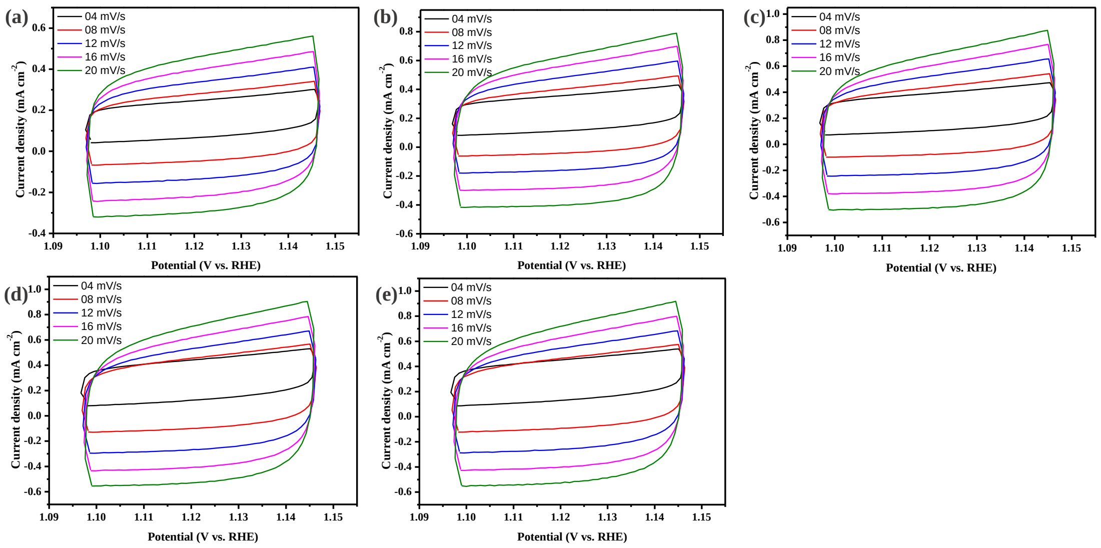

Figure S6. Cyclic voltammogram curves in double layer region at scan rates of 4, 8, 12, 16, 20 $mV\cdot s^{-1}$ of NiMo@VG@CC-AC at the different number of deposition cycles in 1 M KOH electrolyte. (a) 500 cycles, (b) 1700 cycles, (c) 2900 cycles, (d) 4100 cycles, and (e) 5300 cycles.

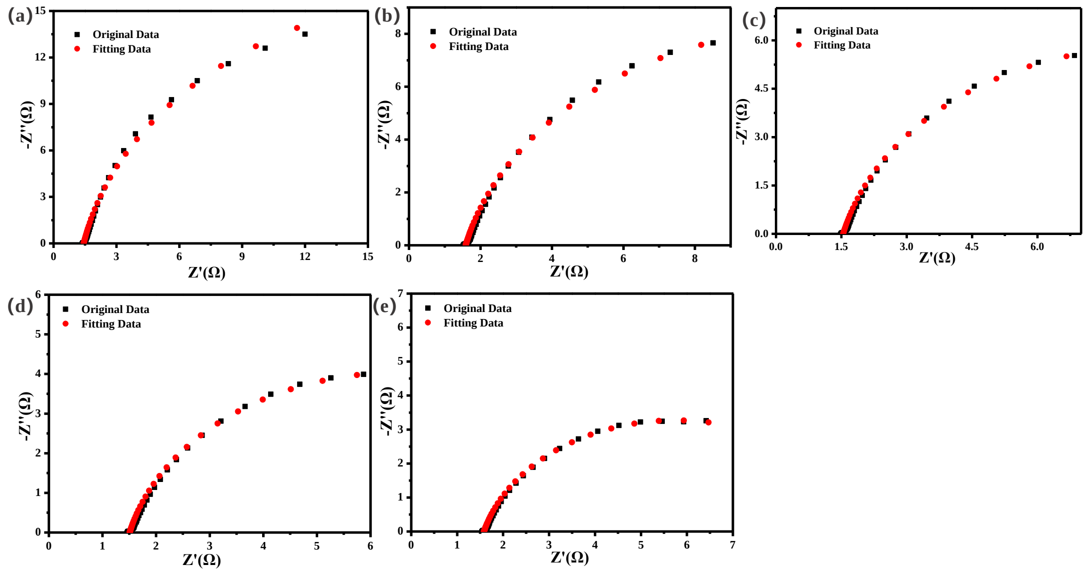

Figure S7. Nyquist plots at the overpotential of 10 mV of NiMo@VG@CC-AC at the different number of deposition cycles in 1 M KOH electrolyte. (a) 500 cycles, (b) 1700 cycles, (c) 2900 cycles, (d) 4100 cycles, and (e) 5300 cycles.

Table S1: Impedance circuit diagram fitting parameters of NiMo@VG@CC-AC with different deposition cycles

Deposition Cycles	R_{Ω}	$R_{\Omega}+R_{ct}$	CPE

500	1.495	40.06	0.01711
1700	1.711	22.03	0.01888
2900	1.705	16	0.02324
4100	1.733	11.64	0.02631
5300	1.886	9.942	0.02767

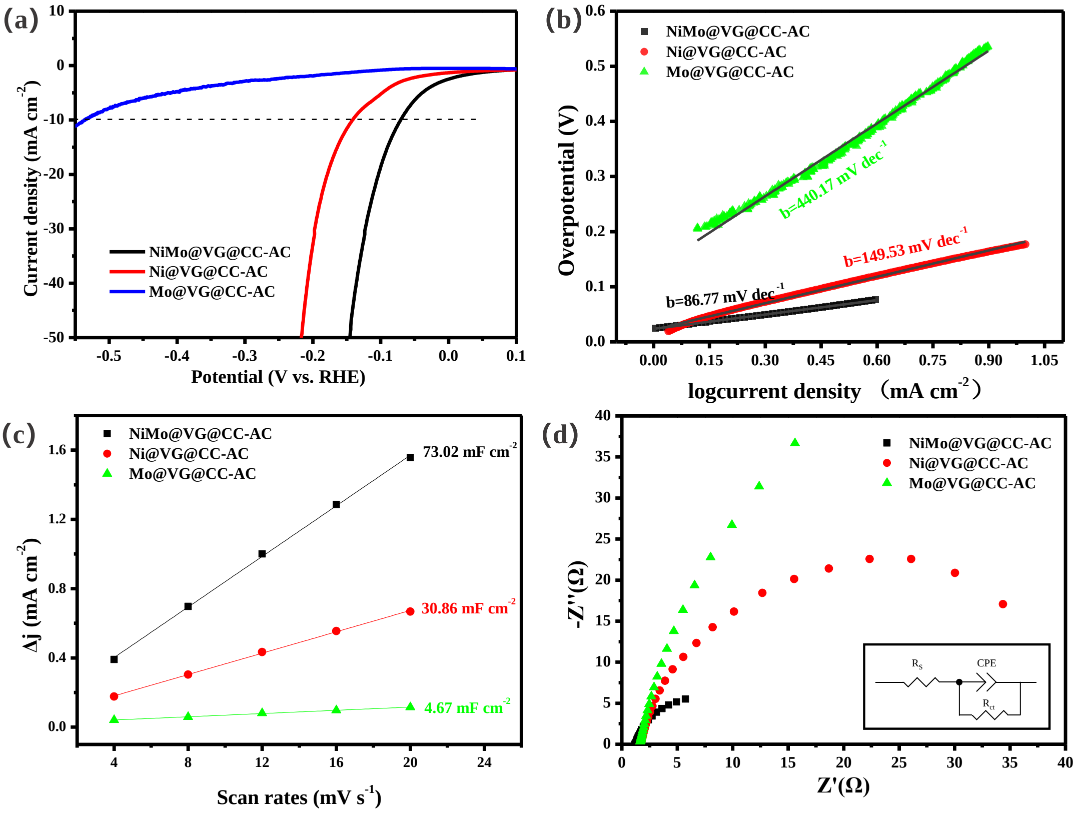

Figure S8. Electrochemical performance of composite catalysts in 1 M KOH electrolyte. (a) HER polarization curves; (b) Corresponding Tafel plots; (c) Calculated double layer capacitance (Cdl) values; (d) Nyquist plots at the overpotential of 10 mV.

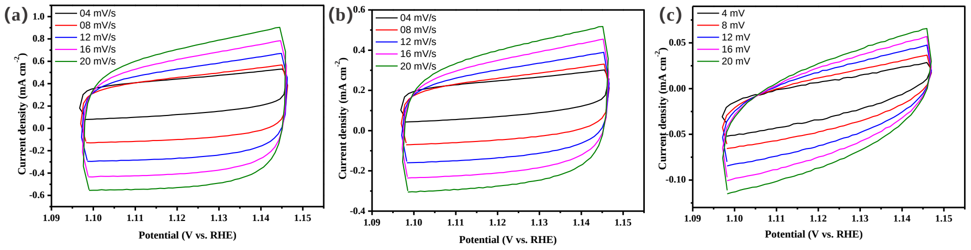

Figure S9. Cyclic voltammogram curves in double layer region at scan rates of 4, 8, 12, 16, 20 mV·s^{-1} of composite catalysts in 1 M KOH electrolyte. (a) NiMo@VG@CC-AC, (b) Ni@VG@CC-AC, (c) Mo@VG@CC-AC.

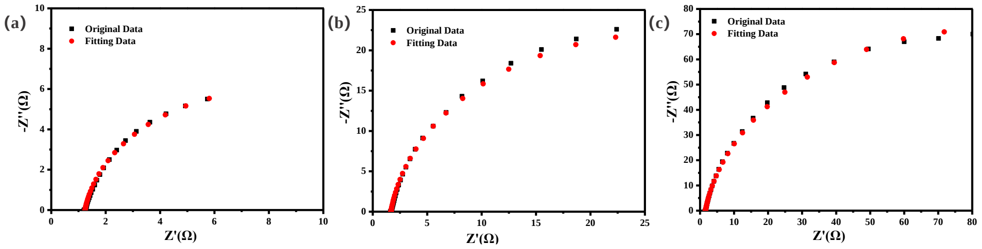

Figure S10. Nyquist plots of composite catalysts in 1 M KOH electrolyte. (a) NiMo@VG@CC-AC, (b) Ni@VG@CC-AC, (c) Mo@VG@CC-AC.

Table S2: Impedance circuit diagram fitting parameters of different composite catalysts

Catalysts	R_Ω	R_Ω+R_{ct}	CPE
NiMo@VG@CC-AC	1.356	14.91	0.03453
Ni@VG@CC-AC	1.732	51.56	0.005022
Mo@VG@CC-AC	1.604	167.04	0.002242

Table S3: Performance comparison of NiMo-based catalysts on various substrates

Catalyst	Electrolyte	Overpotential @10 mV cm^{-2} (mV)	Tafel slope (mV dec^{-1})	Substrate	Reference
NiMo@VG@CC-AC	1 M KOH	71	86	VG@CC-AC	This work
NiMo-Mo_2C/C	0.5 M H_2SO_4	90(@1 mV cm^{-2})	76	Glassy carbon	[44]
Mo-NiMo/NF	1 M KOH	71	104	Nickel foam	[45]
Mo-NiS_x/NF	1 M KOH	136	88	Nickel foam	[46]
$Ni_{0.5}Mo_{0.5}S_x$	1 M KOH	203	78	Glassy carbon	[47]
$Ni_{0.4}Mo_{0.6}$	1 M KOH	72	65	Ti foil	[48]
$MoNi_4$-minor MoC	1 M KOH	69	66	Graphene	[49]
$MoNi_4$	1 M KOH	115	64	Cu foam	[50]

Ni-Mo-Ni $(OH)_2$	1 M KOH	132	134	Carbon cloth	[51]
Ni-Mo-P	1 M KOH	276	76	Nickel foam	[52]
Ni_4Mo	1 M KOH	92	76	Ti foil	[53]
N-NiMoS	1 M KOH	68	86	Nickel foam	[54]
$NiMoP_2$	1 M KOH	144	80	Ti foil	[55]

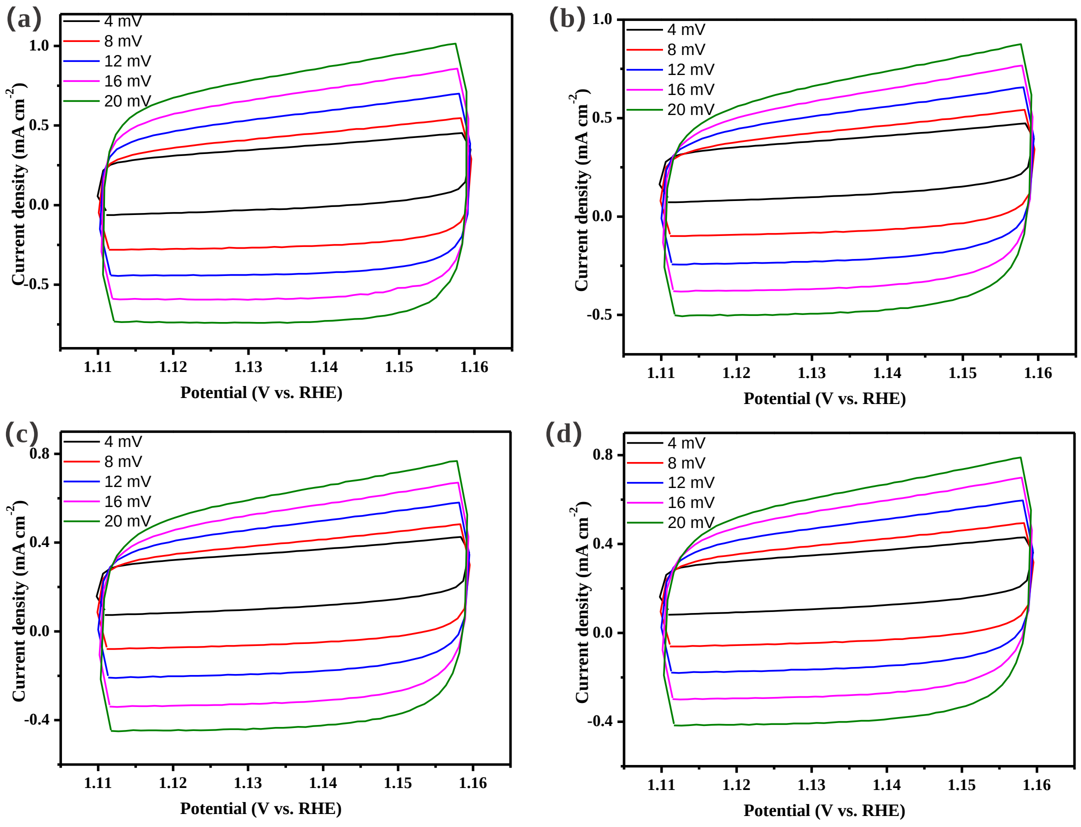

Figure S11. Cyclic voltammogram curves in double layer region at scan rates of 4, 8, 12, 16, 20 mV·s^{-1} of composite catalysts in 1 M KOH electrolyte. (a) NiMo@VG@CC-AC, (b) NiMo@VG@CC-PL, (c) NiMo@CC-AC, (d) NiMo@CC-PL.

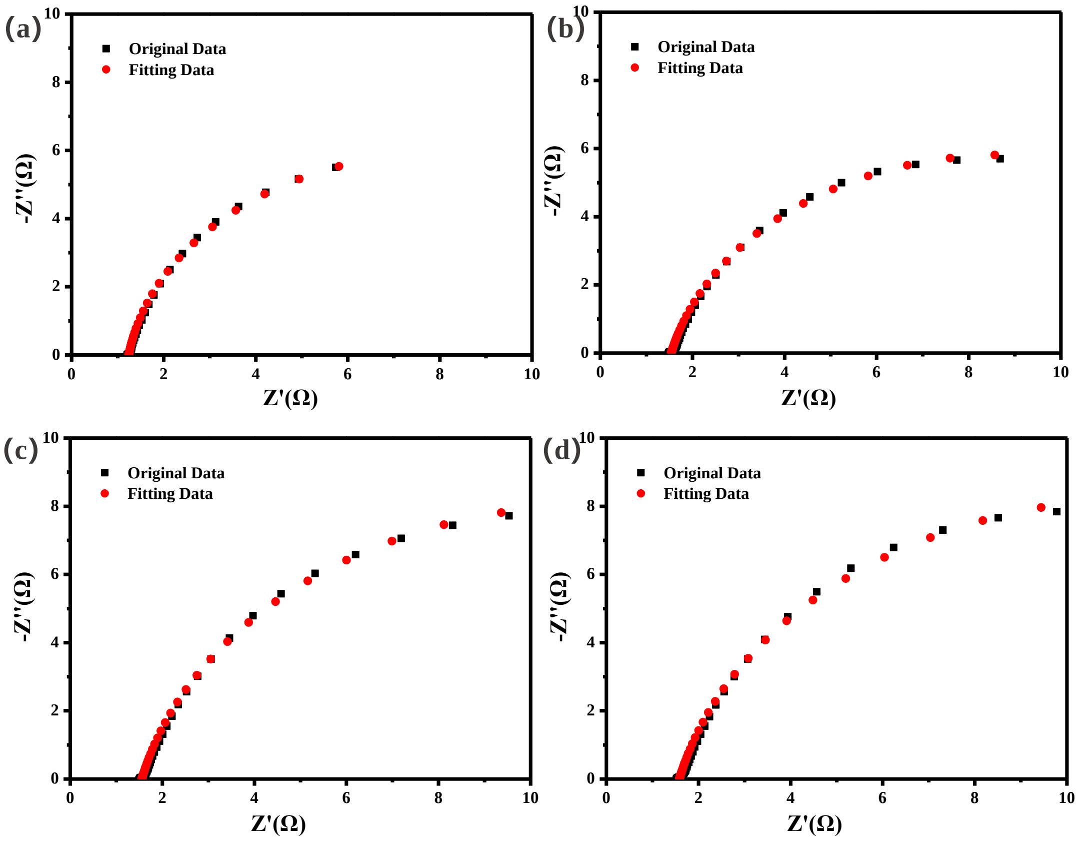

Figure S12. Nyquist plots of composite catalysts in 1 M KOH electrolyte. (a) NiMo@VG@CC-AC, (b) NiMo@VG@CC-PL, (c) NiMo@CC-AC, (d) NiMo@CC-PL.

Table S4: Impedance circuit diagram fitting parameters of NiMo alloy catalysts on various substrates

Catalysts	R_{Ω}	$R_{\Omega}+R_{ct}$	CPE
NiMo@VG@CC-AC	1.356	14.91	0.03453
NiMo@VG@CC-PL	1.705	16	0.02324
NiMo@CC-AC	1.679	21.5	0.02236
NiMo@CC-PL	1.711	22.03	0.01888
